# Supplementary material for: Gluing GAP to RAS Mutants: A New Approach to an Old Problem in Cancer Drug Development
Source: Int J Mol Sci. 2024 Feb 22;25(5):2572. doi: 10.3390/ijms25052572 (PMC10932042; doi:10.3390/ijms25052572)

Gluing GAP to RAS Mutants: A New Approach to an Old Problem in Cancer Drug Development

Supporting material: Table S1

Supporting material: Supplementary Figure S1

Ivan Ranđelović [1], Kinga Nyíri [2,3], Gergely Koppány [2,3], Marcell Baranyi [1,4], József Tóvári [5], Attila Kigyós [1], József Tímár [4], Beáta G. Vértessy [2,3]* and Vince Grolmusz [6,7]*

[1]KINETO Lab Ltd., Budapest, 1037, Hungary
[2]Laboratory of Genome Metabolism and Repair, Institute of Molecular Life Sciences, Research Centre for Natural Sciences, Hungarian Research Network, Budapest, 1117, Hungary
[3]Department of Applied Biotechnology and Food Science, BME Budapest University of Technology and Economics, Budapest, 1111, Hungary
[4]Department of Pathology, Forensic and Insurance Medicine, Semmelweis University, Budapest, 1091, Hungary
[5]Department of Experimental Pharmacology and the National Tumor Biology Laboratory, National Institute of Oncology, Budapest, 1122, Hungary
[6]Mathematical Institute of Eötvös Loránd University,1117 Budapest Hungary
[7]Uratim Ltd., Budapest, 1118, Hungary
*corresponding authors: Vince Grolmusz (grolmusz@pitgroup.org) and Beáta G. Vértessy (vertessy.beata@ttk.hu).

Supplementary Table S1: The fifteen experimentally examined molecules, with ZINC numbers, suppliers, catalogue numbers, structures and molecular weights are listed here. The molecules are referred by their numbers in the first column in the main text. The ZINC numbers refer to the https://zinc.docking.org resource.

ID in main text	ZINC ID	Supplier<br>Catalogue number	Structure	MW (g/mol)
1	72410037	ChemBridge<br>29004287	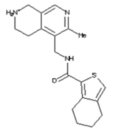	342
2	23339127	ChemBridge<br>43533033	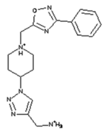	341
3	97632085	ChemBridge<br>59994951	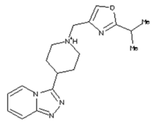	326
4	65383408	ChemBridge<br>27105214	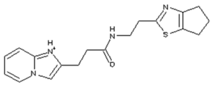	341
5	91526788	ChemBridge<br>37041256	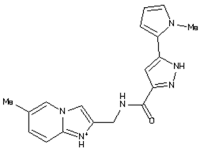	335
6	72419308	ChemBridge<br>16262498	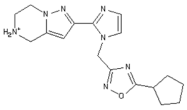	340
7	77385938	ChemBridge<br>42971154	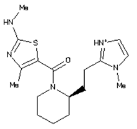	348
8	72407267	ChemBridge<br>11188583	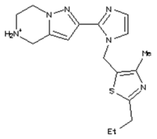	343
9	348992	ChemDiv<br>0263-0451	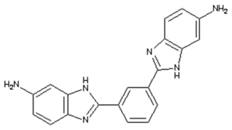	340
10	97201518	ENAMINE<br>Z991000544	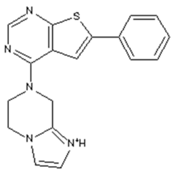	333

11	89701933	ENAMINE<br>Z1170089564	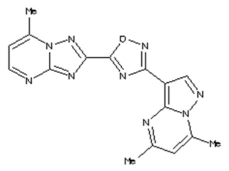	347
12	68640014	ENAMINE<br>Z913154298	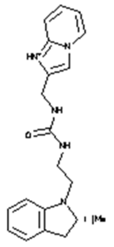	350
13	72278988	ENAMINE<br>Z985662646	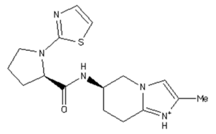	332
14	96231403	InterBioScreen<br>STOCK7S-46707	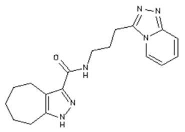	338
15	96119804	Life Chemicals<br>F6413-0713	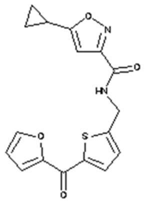	342

Supplementary Figure S1. Differential scanning fluorimetry (DSF) analysis of binding of compound 14 to GAP and $KRAS^{G12D}$ proteins. Curves show the average data from three independent experiments, run in triplicates. Melting points are shown in Table 4 in the main text.

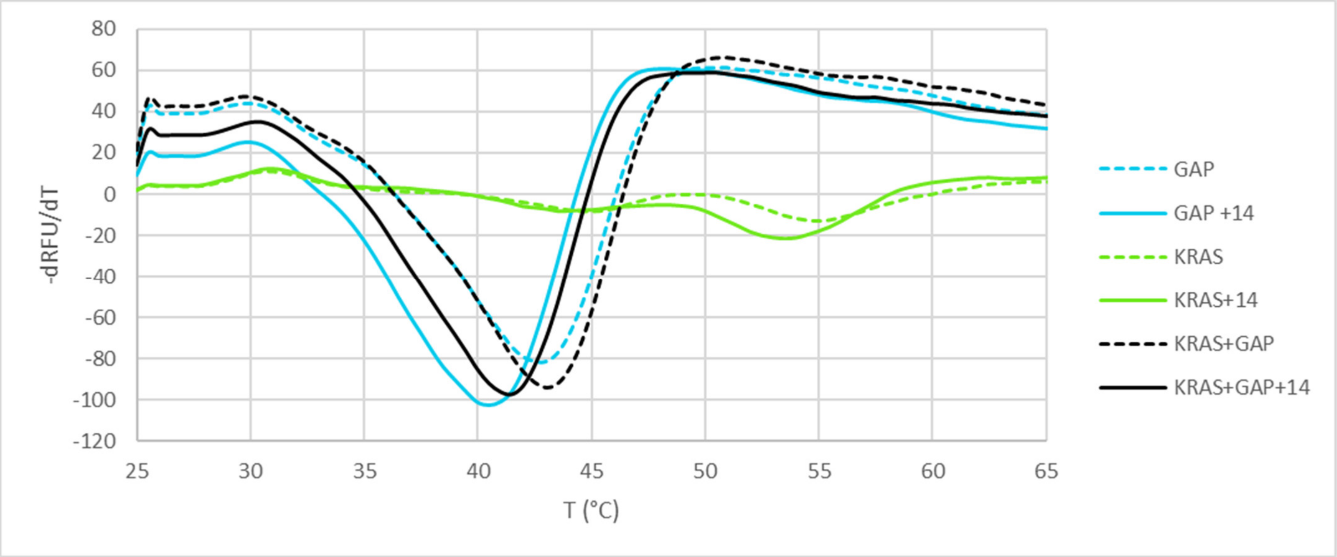

Supplement: Supplementary file 1 [file ijms-25-02572-s001.zip › ijms-2807296-supplementary_revised.pdf]
